# Effectiveness of tixagevimab/cilgavimab in patients with hematological malignancies as a pre-exposure prophylaxis to prevent severe COVID-19: a Czech retrospective multicenter study

**DOI:** 10.1007/s00277-023-05572-0

**Published:** 2023-12-14

**Authors:** Ivo Demel, David Skopal, Eliška Šafránková, Petra Rozsívalová, Pavel Jindra, Jiří Šrámek, Adéla Turková, Jan Vydra, Klára Labská, Jana Vedrová, Martin Čerňan, Tomáš Szotkowski, Heidi Móciková, Lenka Hynková, Ondrej Šušol, Ingrid Kováčová, David Belada, Roman Hájek

**Affiliations:** 1 Department of Haematooncology, University Hospital Ostrava, 17. Listopadu 1790/5, 708 52, Ostrava, Czech Republic; 2 Faculty of Medicine, University of Ostrava, Ostrava, Czech Republic; 3 4th Department of Internal Medicine - Haematology, Hospital and Faculty of Medicine, Charles University, Hradec Kralove, Czech Republic; 4 Hospital Pharmacy, University Hospital Hradec Kralove, Hradec Kralove, Czech Republic; 5 Department of Social and Clinical Pharmacy, Faculty of Pharmacy in Hradec Kralove, Charles University, Hradec Kralove, Czech Republic; 6 Department of Haematology & Oncology, University Hospital Pilsen, Pilsen, Czech Republic; 7 Department of Histology and Embryology, Faculty of Medicine, Pilsen, Czech Republic; 8 Institute of Haematology and Blood Transfusion, Prague, Czech Republic; 9 Department of Haemato-Oncology, Faculty of Medicine and Dentistry, Palacky University and University Hospital Olomouc, Olomouc, Czech Republic; 10 Department of Internal Medicine and Haematology, Faculty Hospital Kralovske Vinohrady, Prague, Czech Republic

**Keywords:** SARS-CoV-2, COVID-19, Tixagevimab/cilgavimab, Hematology malignancy

## Abstract

Despite lower virulence, the omicron variant of severe acute respiratory syndrome coronavirus 2 (SARS-CoV-2) that causes coronavirus disease 2019 (COVID-19) still poses a relevant threat for immunocompromised patients. A retrospective multicentric study was conducted to evaluate the efficacy of pre-exposure prophylaxis with tixagevimab/cilgavimab (Evusheld) with a 6-month follow-up for preventing severe COVID-19 in adult patients with hematology malignancy. Among the 606 patients in the cohort, 96 (16%) contracted COVID-19 with a median of 98.5 days after Evusheld administration. A total of 75% of patients had asymptomatic or mild severity of COVID-19, while just 25% of patients with SARS-CoV-2 positivity had to be hospitalized. Two patients (2%) died directly, and one patient (1%) in association with COVID-19. Eight patients (1.3%) of every cohort experienced adverse events related to Evusheld, mostly grade 1 and of reversible character. It was found that complete vaccination status or positive seroconversion was not associated with lower risk of COVID-19 infection. Previous treatment with an anti-CD20 monoclonal antibody was associated with higher rates of COVID-19, while previous treatment with anti-CD38 monoclonal antibody was not, as was the case for recipients of hematopoietic stem cell transplantation or CAR-T cell therapy. Presence of other comorbidities was not associated with more severe COVID-19. The results support the growing evidence for Evusheld’s efficacy against severe COVID-19 in patients with hematology malignancies.

## Introduction

Since the outbreak of the severe acute respiratory syndrome coronavirus 2 (SARS-CoV-2) pandemic at the end of 2019, causing the coronavirus disease 2019 (COVID-19), several specific anti-SARS-CoV-2 pharmacotherapy approaches have been introduced to clinical practice, including vaccination, monoclonal antibodies (mAbs), and antiviral drugs. Evusheld**™** (AstraZeneca, AB, Södertälje, Sweden) is a combination of two long-acting antibodies, tixagevimab and cilgavimab, which are two recombinant human IgG1κ mAbs with amino acid substitutions to extend antibody half-life. They can simultaneously bind to distinct regions of the receptor binding domain of SARS-CoV-2 spike protein, thus blocking its interaction with human ACE2, a receptor required for virus attachment. Evusheld has been approved for pre-exposure prophylaxis of COVID-19 in the European Union (EU) since 28 March 2022, as it significantly reduced the risk of developing symptomatic COVID-19, with protection lasting at least 6 months [1]. Later that year, Evusheld was approved in the EU for the treatment of COVID-19, as it provided protection against progression to severe COVID-19 compared to a placebo [2]. However, in the former registration PROVENT study, recipients of immunosuppressive therapy or immunocompromised patients represented only 3.6% of all analyzed participants. This paper presents retrospective real-world data from 606 high-risk hemato-oncological patients who received Evusheld as a pre-exposure prophylaxis to prevent the development of symptomatic COVID-19 in a full 6-month protective period.

## Methods and goals

This multicentric retrospective study included adult patients with hemato-oncological disease who were given tixagevimab/cilgavimab (Evusheld) in a total dose of 300 mg (150 mg of tixagevimab and 150 mg of cilgavimab, administered as two separate, sequential mostly intramuscular injections) across six hematological centers in the Czech Republic. After administration, each patient had to finish a 6-month follow-up to complete the study. Subjects who were lost from the follow-up or died of other causes than COVID-19 were excluded from the final analysis. Patients who died in association with COVID-19 were included. Evusheld prophylaxis administration was available from 3 March 2022, following temporary regulatory authorization by the Czech Republic Ministry of Health. Disease severity was assessed according to adapted definitions [3]. Data were obtained from source medical documentation covering patient characteristics, comorbidities, and hematological disease. Data including the history of vaccination against SARS-CoV-2 as well as positive testing for SARS-CoV-2 were obtained from Registers of the Ministry of Health. Data covering the course of the COVID-19 disease, including severity, other anti-SARS-CoV-2 medications, and outcome, were obtained from source documentation and from direct contact with the patient in some ambiguous cases.

Vaccination against SARS-CoV-2 was performed independently of this trial, with the regulatory authorization by the Czech Ministry of Health. A completed vaccination scheme was defined as a basic set of first and second doses of Comirnaty, Spikevax, and Vaxzevria vaccines, followed by at least one boosting dose of Comirnaty or Spikevax vaccine. For the Janssen vaccine, one basic dose is followed by at least one boosting dose of Comirnaty or Spikevax. An incomplete vaccination scheme was defined as two and less doses of any vaccine (except Janssen). Testing for antibody detection against SARS-CoV-2 was performed independently of this trial and in just one center (Ostrava). The methodology was described by Šušol et al. [4]. Briefly, serum samples were tested for the detection of IgG, IgM, and IgA anti-S1/S2 antibodies (immunoglobulins antibodies, IgAbs) to SARS-CoV-2 using a commercial enzyme-linked immunosorbent assay (ELISA) and for the detection of neutralizing antibodies using the inhouse in vivo virus neutralization test (VNT) against the omicron variant viruses. The quantity of the antibodies was also measured utilizing the binding antibody unit (BAU) per ml as a World Health Organization standard for reference. For IgAbs, the signal-to-cut-off ratio was calculated and values < 0.9 were regarded as negative, ≥ 0.9 to < 1.1 as borderline, and ≥ 1.1 as positive seroconversion. For VNT, the positivity was determined as a titer of 20 or higher. For BAU, the positivity was determined as s titer of ≥ 21.8 BAU/ml.

The research was conducted with respect to relevant guidelines and regulations with project approval by the local ethics committee of each hospital involved. All patients involved in this study signed an informed consent form, allowing health staff to see their medical documentation.

Basic statistical methods describing absolute and relative frequency for categorical variables, median and IQR for continuous variables, respectively, were employed. Categorical parameter relations were evaluated using Fisher’s exact test with *p* = 0.05 as a statistical significance level.

The primary outcomes were (i) incidence of SARS-CoV-2, verified with reverse transcription polymerase chain reaction (RT‐PCR) or antigen testing, in the study population during 6 months follow-up after administration of Evusheld, and its severity followed by the outcome of these patients, and (ii) evaluation of adverse events after Evusheld administration.

Secondary outcomes were as follows: (i) evaluation of the so-called double pre-exposition prophylaxis, i.e., incidence of COVID-19 in patients that completed a full vaccination scheme against SARS-CoV-2 or reached positive seroconversion after previously having experienced COVID-19 or after vaccination against SARS-CoV-2; (ii) probability of COVID-19 in patients with anti-CD20 or anti-CD38 therapy up to 6 months before Evusheld or the probability of COVID-19 in CAR-T therapy recipients or patients who underwent hematopoietic stem cell transplantation (HSCT) up to 2 years before Evusheld administration or during the 6-month follow-up after Evusheld; (iii) an evaluation of the severity of COVID-19 in patients with additional risk factors.

## Results

**Fig. 1:**
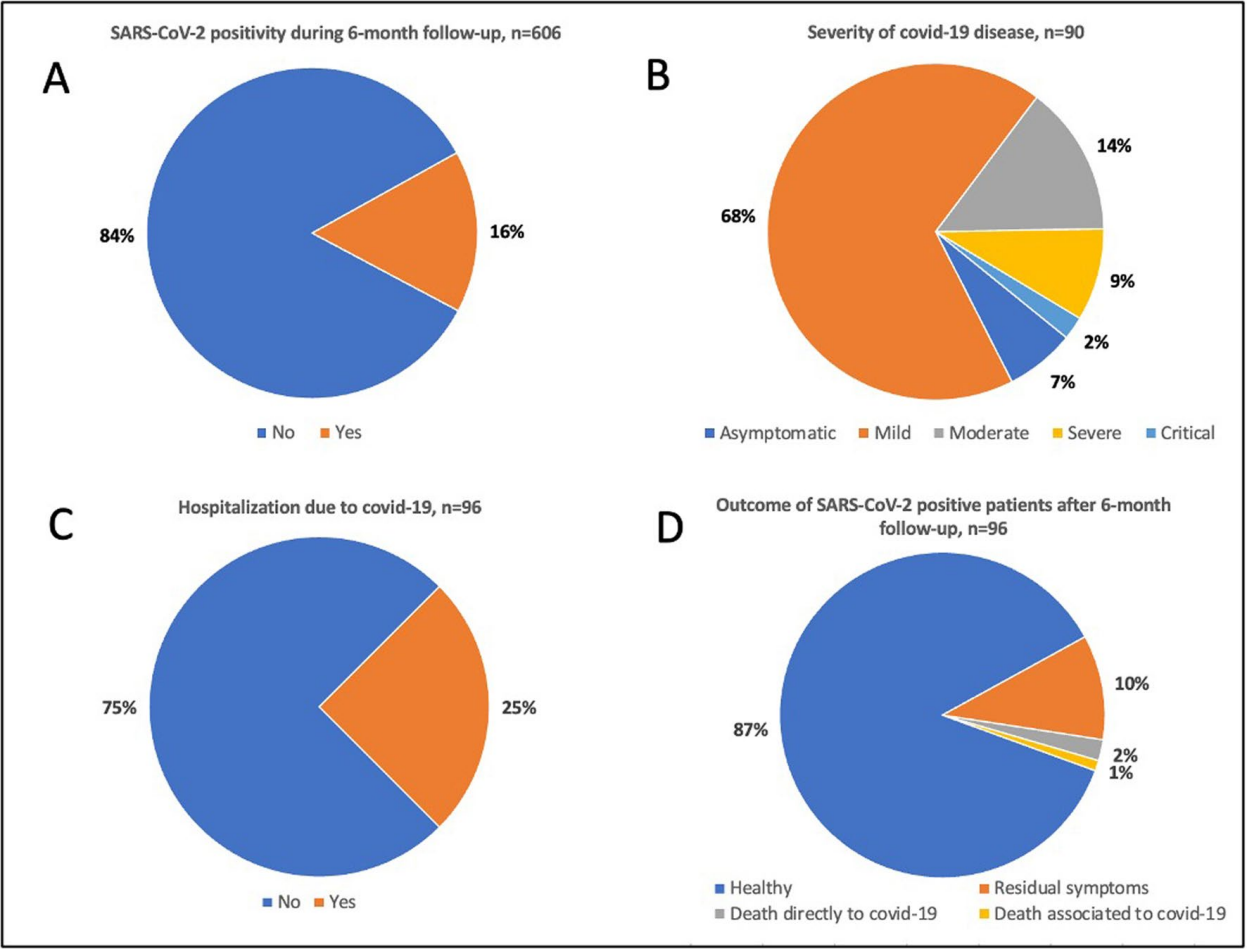
The results of the primary outcomes

Between April 8, 2022, and September 30, 2022, a total of 641 patients were enrolled in the study. Of these, 35 patients were excluded from the study because they did not complete the 6-month follow-up due to death from underlying hematology disease or death by a cause other than COVID-19 or were lost to the follow-up. Overall, 606 patients were included in the final analysis. The study population’s characteristics are represented in Table 1. With the median age being 65.5 years (IQR 15.8) at the time of Evusheld administration, multiple myeloma, indolent and aggressive non-Hodgkin lymphoma, and chronic lymphocytic leukemia were the most frequent underlying diagnoses (79%, 478/606). Most of the patients (76%, 441/579) were actively treated at the time of Evusheld administration (i.e., no more than 30 days since the last hematologic treatment). The results of the primary outcomes are summarized in Fig. 1 and Table 2. Out of 606 patients, 96 (16%) patients had a SARS-CoV-2 positive test during the 6-month follow-up after Evusheld administration. The median time for a positive test since Evusheld administration was 98.5 days (IQR 91.0). The COVID-19 severity was evaluated in 90/96 SARS-CoV-2 positive patients. Of these, 7% (6/90) had an asymptomatic, 68% (61/90) mild, 14% (13/90) moderate, 9% (8/90) severe, and 2% (2/90) critical clinical course of COVID-19. Thus, the total incidence of symptomatic COVID-19 illness was 13.8% (84/606). A total of 24 out of 96 (25%) patients with COVID-19 had to be hospitalized. The majority of SARS-CoV-2 positive patients had undergone specific antiviral treatment, such as remdesivir (43%, 41/96), molnupiravir (35%, 34/96), nitrmatrelvir/ritonavir (19%, 18/96), or a combination therapy (6%, 6/96). In addition to the first COVID-19 positive episode, 10 out of 96 patients experienced re-infection or reactivation with SARS-CoV-2. Of these, 4 had mild, 4 had moderate, and 2 had a severe course of COVID-19 reinfection. The outcome of the 96 SARS-CoV-2 positive patients was mostly favorable, as 87% (83/96) of the patients with a COVID-19 infection completely recovered at the end of the 6-month follow-up and 10% (10/96) of the patients were experiencing residual symptoms of COVID-19, with dyspnea, fatigue, and deterioration of smell being the most common. Two patients out of 96 (2%) passed away directly in connection to COVID-19. Of these patients, one was an 82-year-old female treated with relapse/refractory multiple myeloma that had been treated over 3 years (last line therapy included anti-B-cell maturation antigen bispecific antibody), with a medical history of colorectal carcinoma post-hemicolectomy and adjuvant chemotherapy, and, moreover, with a history of arterial hypertension and chronic kidney disease. The second patient was a 61-year-old female patient with primary myelofibrosis who underwent allogeneic SCT nearly a year before COVID-19 diagnosis. She had multiple comorbidities, of which the most severe were asthma and liver cirrhosis Child–Pugh A. One death was assessed as a complication associated with COVID-19. It was a 60-year-old patient with acute myeloid leukemia, in complete remission nearly 1 year after allogeneic SCT, with chronic asthma, that succumbed to superseding bilateral bacterial pneumonia shortly after COVID-19 pneumonia. Overall, out of the 606 patients involved in the final analysis, 3 (0.5%) patients died due to COVID-19.

**Table 1:** Patient’s characteristics

Number of patients	606
Sex	
Male	328 (54%)
Female	278 (46%)
Age at the time of Evusheld administration, years, median (IQR)	65.5 (15.8)
Manner of administration	
Intramuscular	604 (97.7%)
Intravenous	2 (0.3%)
Comorbidities	
No	109 (18%)
Yes	497 (82%)
• Arterial hypertension	329 (66%)
• Chronic renal disease with eGFR < 60 ml/min/1.73 m^2^	104 (21%)
• Diabetes mellitus	97 (20%)
• Ischemic heart disease	55 (11%)
• Obesity with BMI > 30	143/496 (29%)
• Smoking	78/472 (17%)
• Chronic pulmonary disease	97 (20%)
Underlying hematological disease at the baseline	
Multiple myeloma and other monoclonal gammopathies	154 (25%)
NHL indolent	138 (23%)
NHL aggressive	100 (17%)
Chronic lymphocytic leukemia	86 (14%)
Acute leukemia	63 (10.3%)
Myelodysplastic syndrome	29 (4.8%)
Myeloproliferative neoplasm	10 (1.6%)
Hodgkin lymphoma	9 (1.5%)
Other	17 (2.8%)
Time since hematological diagnosis, years, median (IQR)	1.7 (4.3)
Treatment	
Active treatment (< 30 days since last medication)	441/579 (76%)
Observation	138/579 (24%)
Anti-CD20 therapy < 6 months before Evusheld	236 (39%)
Anti-CD38 therapy < 6 months before Evusheld	73 (12%)
HSCT or CAR-T therapy < 2 years before Evusheld	118 (19%)
Allogeneic SCT	68 (11.2%)
Autologous SCT	45 (7.4%)
CAR-T	5 (0.8%)
Time since HSCT or CAR-T therapy, days, median (IQR)	56.5 (234.8)
HSCT or CAR-T therapy after Evusheld	84 (14%)
Allogeneic SCT	34 (5.6%)
Autologous SCT	43 (7.1)
CAR-T	7 (1.2%)
Time since Evusheld administration, days, median (IQR)	25.0 (95.8)

Values are the number (%) of patients unless stated otherwise. *IQR*, interquartile range; *BMI*, body mass index; *NHL*, non-Hodgkin lymphoma; *HSCT*, hematopoietic stem cell transplantation; *SCT*, stem cell transplantation; *CAR-T*, chimeric antigen receptor therapy

**Table 2:** Characteristics of hematology patients with breakthrough COVID-19 disease after pre-exposure prophylaxis with Evusheld

Number of patients	96
Age at the time of SARS-CoV-2 positivity, years, *median (IQR)*	65.4 (17.8)
Time since Evusheld to SARS-CoV-2 positivity, days, median (IQR)	98.5 (91.0)
Underlying hematological disease of SARS-CoV-2 positive patients	
• NHL indolent	24 25%)
• NHL aggressive	24 25%)
• Multiple myeloma and other monoclonal gammopathies	19 20%)
• Acute leukemia	11 11%)
• Chronic lymphocytic leukemia	9 (9%)
• Myeloproliferative neoplasm	5 (5%)
• Myelodysplastic syndrome	3 (3%)
• Other	1 (1%)
Treatment	
• Active treatment (< 30 days since last medication)	74/93 (80%)
• Observation	19/93 (20%)
Anti-CD20 therapy < 6 months before Evusheld	47 (49%)
Anti-CD38 therapy < 6 months before Evusheld	11 (12%)
HSCT or CAR-T therapy < 2 years before Evusheld or after Evusheld	38 (31%)
Positive IgAbs, VNT or BAU	7 (7%)
At least one dose of vaccination	83 (86%)
Complete vaccination scheme (i.e., at least three doses*)	53 (55%)
Comorbidities	
No	14 (15%)
Yes	82 85%)
• Arterial hypertension	49 51%)
• Chronic renal disease with eGFR < 60ml/min/1.73m^2^	16 17%)
• Diabetes mellitus	20 (21%)
• Ischemic heart disease	7 (7%)
• Obesity with BMI > 30	19 (20%)
• Smoking	13 (14%)
• Chronic pulmonary disease	21 (22%)

Values are the number (%) of patients unless stated otherwise. *IQR*, interquartile range; *NHL*, non-Hodgkin lymphoma; *HSCT*, hematopoietic stem cell transplantation; *SCT*, stem cell transplantation; *CAR-T*, chimeric antigen receptor therapy; *IgAbs*, immunoglobulins antibodies; *VNT*, virus neutralization test; *BAU*, binding antibody units; *BMI*, body mass index; *eGFR*, estimated glomerular filtration rate

*At least two doses of JCOVDEN vaccine

Regarding the safety profile, only 1.3% (8/606) of patients experienced an adverse event (AE) related to Evusheld prophylaxis (Table 3). Of these, nausea was observed in 3 out of 8 patients, exanthema was observed in 3 out of 8 patients, fever was observed in 2 out of 8 patients, faintness was observed in 2 out of 8 patients, and diplopia and painfulness at the site of Evusheld administration was observed in 1 out of 8 patients. The median duration of AE was 6.5 days (IQR 5.8). Most of the AEs were only mild and resolved spontaneously or after symptomatic treatment (7/8); thus, they were evaluated as grade 1 according to Common Terminology Criteria for Adverse Events version 5 (CTCAEv5). Diplopia due to a suspected lesion of the abducent nerve (grade 2 according to CTCAEv5) occurred 5 days after Evusheld administration. However, the patient’s neurological state deteriorated over time, and 1 month later, he was diagnosed with fungal meningoencephalitis caused by *Candida krusei* (PCR-positive cerebrospinal fluid). Thus, the diplopia most likely had a para-infectious etiology.

**Table 3:** Adverse events. Grading is according to the Common Terminology Criteria for Adverse Events version 5

*N*	606
Adverse events	8/606 (1.3%)
Grade 1	
• Nausea	3/8 (37.5%)
• Exanthema	3/8 (37.5%)
• Fever	2/8 (25%)
• Faintness	2/8 (25%)
• Painfulness at the site of administration	1/8 (12.5%)
Grade 2	
• Diplopia	1/8 (12.5%)
Duration of adverse event, days, median (IQR)	6.5 (5.8)

Values are the number (%) of patients unless stated otherwise

As a secondary endpoint, an evaluation was made as to whether the history of a complete vaccination scheme against SARS-CoV-2 before the Evusheld administration had an additional protective effect. The same was applied to a subpopulation of patients who had measurable levels of anti-SARS-CoV-2 antibodies postvaccination or due to a history of COVID-19 before Evusheld administration. An overview of COVID-19 and vaccination history in the study population is shown in Table 4. A total of 48.8% (296/606) of patients have experienced COVID-19 before Evusheld administration, most of them with asymptomatic or mild severity (72.4%, 213/296). The median time of COVID-19 diagnosis before Evusheld was 224 days (IQR 381.5). In addition, 22% (65/295) of patients had experienced a reinfection or reactivation with COVID-19 before the administration of Evusheld. Regarding vaccination, 86% (529/606) of patients received at least one dose of any vaccine, but only 59.6% (361/606) of patients fully completed vaccination scheme (see “Methods and goals”). Most of the patients were vaccinated with the first dose in the first half of year 2021 (85%, 444/525). One hundred four, 103, and 101 patients were tested for IgAbs, VNT, or BAU antibodies, respectively. The presence of antibodies (i.e., termed as positive seroconversion) was similar for each entity—62% for IgAbs, 63% for VNT, and 64% for BAU. Using the calculation of relative frequencies of COVID-19 throughout the 6-month follow-up, the results were negative, with a *p*-value of 0.4 for a complete vaccination scheme, and > 0.9 for antibodies, respectively. The same result (*p* =  > 0.9) was observed for both single antibody positivity and triple antibody positivity. Overall, there was no observation that patients with a complete vaccination scheme or positive IgAbs, VNT, or BAU titers of antibodies had a lower incidence of COVID-19 disease after the prophylactic administration of Evusheld (Table 5).

**Table 4:** Status of COVID-19 and vaccination before Evusheld

Number of patients	606
COVID-19 before Evusheld	296 (48.8%)
Severity of COVID-19 before Evusheld	
• Asymptomatic	25 (8.4%)
• Mild	188 (64%)
• Moderate	52 (18%)
• Severe	22 (7.4%)
• Critical	8 (2.7%)
• Unknown	1 (0.3%)
Time of COVID-19 infection before Evusheld, days, median (IQR)	224 (381.5)
Reinfection with COVID-19 before Evusheld	65/295 (22%)
Vaccination against SARS-CoV-2 before Evusheld (at least one dose)	529 (86%)
• Comirnaty (Pfizer/BioNTech)	389 (64%)
• Spikevax (Moderna)	100 (17%)
• Vaxzevria (AstraZeneca)	34 (5.6%)
• JCOVDEN (Janssen, J&J)	6 (1%)
• None	77 (13%)
Completed vaccination scheme (at least three doses*)	361 (59.6%)
Incomplete vaccination scheme or no vaccination	245 (40.4%)
Positive IgAbs after vaccination or COVID-19	65/104 (62%)
Positive VNT after vaccination or COVID-19	65/103 (63%)
Positive BAU after vaccination or COVID-19	65/101 (64%)

Values are the number (%) of patients unless stated otherwise. *IgAbs*, immunoglobulins antibodies; *VNT*, virus neutralization test; *BAU*, binding antibody units

*At least two doses of JCOVDEN vaccine

**Table 5:** Secondary outcomes. Relative frequencies of COVID-19 disease throughout the 6-month follow-up after Evusheld administration for a complete and incomplete vaccination scheme, for positive seroconversion, for anti-CD20 therapy, for anti-CD38 therapy, and HSCT or CAR-T therapy

	COVID-19 disease at 6-month follow-up			
	*N*	Yes (*n* = 96)	No (*n* = 510)	*p*-value^1^
Vaccination	606			0.4
Completed vaccination scheme		53 (15%)	308 (85%)	
Incomplete vaccination scheme		43 (18%)	202 (82%)	
Positive IgAbs, VNT, and BAU	96			> 0.9
Yes		7 (12%)	52 (88%)	
No		4 (11%)	33 (89%)	
Anti-CD20 therapy	606			**0.031**
Yes		47 (20%)	189 (80%)	
No		49 (13%)	321 (87%)	
Anti-CD38 therapy	606			> 0.9
Yes		11 (15%)	62 (85%)	
No		85 (16%)	448 (84%)	
HSCT or CAR-T therapy	606			0.2
Yes		38 (19%)	164 (81%)	
No		58 (14%)	346 (86%)	

Bold emphasis underlines that the *p*-value is statistically significant

*IgAbs*, immunoglobulins antibodies; *VNT*, virus neutralization test; *BAU*, binding antibody units; *HSCT*, hematopoietic stem cell transplantation; *CAR-T*, chimeric antigen receptor therapy

^1^Fisher’s exact test

The focus then moved to the highest-risk group of patients. Overall, 39% (236/606) of patients received anti-CD20 therapy, and 12% (73/606) received anti-CD38 therapy up to 6 months before Evusheld administration, respectively. Furthermore, 33% (202/606) patients underwent HSCT (*n* = 195) or CAR-T (*n* = 7) therapy up to 2 years before Evusheld administration or during the 6-month follow-up. Using the calculation of relative frequencies of COVID-19 throughout the 6-month follow-up, the results were positive for the anti-CD20 group of patients (*p* = 0.031), but negative for the anti-CD38 (*p* =  > 0.9) and HSCT/CAR-T group (*p* = 0.2), respectively. Thus, it was observed that patients who received anti-CD20 therapy up to 6 months before Evusheld were at a higher risk of COVID-19 throughout the 6-month follow-up after Evusheld administration (Table 5).

Finally, an assessment was made as to whether the presence of additional comorbidities was a risk factor for worse COVID-19 severity after Evusheld administration. For that, the COVID-19-positive patients were divided into two groups: one with COVID-19 severity ≤ 1 (i.e., asymptomatic and mild) and the second with COVID-19 severity ≥ 2 (i.e., moderate, severe, and critical). Then, the frequencies of these two groups were compared between patients who did or did not present with one of these comorbidities: arterial hypertension, chronic renal insufficiency with eGFR < 60 ml/min/1.73 m^2^, ischemic heart disease, obesity with body mass index (BMI) > 30, history of smoking, or chronic pulmonary disease. Results were negative as none of the observed comorbidity was associated with a statistically significant, more severe clinical course of COVID-19 (Table 6).

**Table 6:** Comorbidities and COVID-19 severity. Relative frequencies of COVID-19 severity in patients with certain comorbidities throughout the 6-month follow-up after Evusheld administration

	COVID-19 disease severity at 6-month follow-up			
	*N*	*Gr*. ≤ 1 (*n* = 67)	*Gr*. ≥ 2 (*n* = 29)	*p*-value^1^
Arterial hypertension	96			0.7
Yes		33 (67%)	16 (33%)	
No		34 (72%)	13 (28%)	
Chronic kidney disease	96			0.4
Yes		13 (81%)	3 (19%)	
No		54 (68%)	26 (32%)	
Diabetes mellitus	96			0.8
Yes		15 (75%)	5 (25%)	
No		52 (68%)	24 (32%)	
Ischemic heart disease	96			0.4
Yes		4 (57%)	3 (43%)	
No		63 (71%)	26 (29%)	
Obesity with BMI > 30	96			0.2
Yes		14 (74%)	5 (26%)	
No		53 (69%)	24 (31%)	
Smoking	95			0.3
Yes		11 (85%)	2 (15%)	
No		55 (67%)	27 (33%)	
Chronic pulmonary disease	96			0.4
Yes		13 (62%)	8 (38%)	
No		54 (72%)	21 (28%)	

*Gr*., grade, *BMI*, body mass index

^1^Fisher’s exact test

## Discussion

The results are not comparable with results from the PROVENT trial (1), where the total incidence of symptomatic SARS-CoV-2 positive illness throughout the 6-month follow-up was much lower (0.2%). Firstly, there was a different overall incidence of COVID-19 in the general population at the time of enrollment for those trials. Second, the overall number of immunocompromised subjects in the PROVENT trial in arm with Evusheld was only 3.6% (124/3460). However, several different studies with hemato-oncological patients have already been published.

One of the first clinical trials in a real-world setting was published by Ocon et al. exploring the efficacy of Evusheld in 203 patients with hematologic malignancies [5]. The proportion of patients treated with anti-CD20 therapy was similar in both our and their study populations (39% vs. 44%). During the median follow-up of 158 days, 9.3% (19/203) of patients developed symptomatic COVID-19, with only one patient requiring hospitalization. These are slightly better results compared to the ones presented here (13.8% [84/606] symptomatic COVID-19, 25% [24/96] hospitalization rate among all COVID-19 positive). A possible explanation of this difference is the administration of a higher dose of 300 mg tixagevimab and 300 mg cilgavimab in the USA as a pre-exposure prophylaxis, which represents a double dosing as opposed to what was permitted in the EU at the time.

Despite a lower severity of infection compared to the delta variant [6], the mortality among hospitalized hemato-oncological patients with omicron variant is still high (16.5%), as shown by data from the EPICOVIDEHA register [7]. In the cohort of hemato-oncological patients with pre-exposure prophylaxis with Evusheld, the overall mortality among all COVID-19-positive patients was considerably lower—only 3%. Later, the EPICOVIDEHA group analyzed 47 hemato-oncological patients with breakthrough COVID-19 infection after Evusheld administration. It showed a higher frequency of more severe (i.e., moderate, severe, and critical) COVID-19 infection than the findings from this study (66% vs. 25%), but similar rates of hospitalization (21.3% vs. 25%) and death (2.1% vs. 2%). In a subsequent matched-paired analysis with a control group without Evusheld administration, a reduction of COVID-19 severity, proportion of hospitalization, and all-cause mortality was reported [8].

Patients undergoing HSCT or recipients of CAR-T therapy are considered to be one of the highest-risk patients regarding COVID-19. However, this data showed that these conditions were not associated with higher incidence of COVID-19. This is in accordance with a retrospective study by Jondreville et al. that reported efficacy of pre-exposure prophylaxis with tixagevimab/cilgavimab in 161 patients after allo-HSCT. With the median follow-up of 105 days, 14% (22/161) of patients developed symptomatic COVID-19 infection, with no severe cases or death occurring [9]. In the present cohort, the numbers were similar as 19% (38/202) of patients with HSCT or CAR-T therapy experienced COVID-19, with one fatal case described above.

Patients with multiple myeloma, especially those treated with anti-CD38 mAbs, are considered high risk for breakthrough COVID-19 due to suboptimal antibody response following vaccination and underlying immune deficiency from the disease itself [10, 11]. However, we did not observe higher rates of COVID-19 among recipients of anti-CD38 treatment. Similarly, a subpopulation of patients with B-cell malignancies is at increased risk of morbidity and mortality due to the use of B-cell-depleting agents as well as low seroconversion after vaccination [12, 13]. Indeed, significantly higher rates of COVID-19 infections were observed among recipients of anti-CD20 therapy (20% vs. 13%, *p* = 0.031). These numbers are higher than those published by Davis et al. who reported 11% COVID-19 infections among patients with B-cell malignancies. Of those, two-thirds were recipients of B-cell-depleting therapy [14]. Yet again, the majority of these patients had received a cumulative dose of 300 mg/300 mg tixagevimab/cilgavimab. Thus, for these vulnerable populations, besides vaccination, additional mitigation strategies such as pre-exposure prophylaxis are desirable, at least to reduce the risk of severe course of COVID-19.

Recently, a prospective study evaluated the rate of SARS-CoV-2 infection following pre-exposure prophylaxis with Evusheld in seronegative patients, compared to a cohort of seropositive patients who were only observed or received a fourth vaccine dose [15]. The results were similar with the 3-month cumulative incidence of SARS-CoV-2 infection, being 20% for seronegative with the Evusheld group of patients and 12% for seropositive with the vaccination group of patients (*p* = 0.34). In the present study, there were also no observations of a reduced incidence of COVID-19 in patients who were seropositive versus seronegative or had a complete versus an incomplete vaccination scheme. These results suggest that the presence of antibodies, either from previous SARS-CoV-2 infection or after vaccination, does not provide an additional protective effect when compared to or combined with Evusheld. In fact, primary immunization with two doses of Vaxzevria or Comirnaty vaccines provided limited protection against symptomatic disease caused by the omicron variant, with booster doses substantially increasing protection, but with that protection waning over time [16]. Similar results were observed with first-generation vaccines against the BA.2 subvariant of omicron [17].

Lastly, it is known that patients with certain underlying medical conditions are at a higher risk of experiencing severe outcomes of COVID-19 in the general population, as listed by the Centers for Disease Control and Prevention [18]. An attempt was made to evaluate whether one of these conditions may contribute to a more severe COVID-19 in this cohort. However, a statistically significant difference was not observed for any comorbidity. This may be partly due to the small number of subjects, especially in the COVID-19-positive group. Another possible explanation is that hematological malignancy itself is one of the strongest risk factors for severe COVID-19. On the other hand, Kamboj et al. showed that diabetes and renal failure were independent predictors of severe COVID-19 in a group of Evusheld recipients with hematologic malignancies at the time when resistant variants dominated regionally [19].

The present study confirms the favorable safety profile of Evusheld. We observed a 1.3% (8/606) count of AEs related to Evusheld administration, most of them mild and not requiring intervention or hospitalization. Exanthema (or so-called hypersensitivity) was commonly described in the pivotal trial, as well as injection site pain. Other AEs might be also related to basic hematology disease (faintness due to anemia, for example), treatment (nausea as a part of chemotherapeutic-induced nausea), or infection complications (fever). In addition, one of the AEs, diplopia, was later reassessed as most likely para-infectious etiology. We did not observe any AEs of special interest, nor unexpected longer-term safety signals.

To our knowledge, this is one of the largest presented cohorts of hemato-oncological patients that were given Evusheld as a pre-exposure prophylaxis. Other strengths of the study are the involvement of multiple hematological centers across the Czech Republic with variable hematology diseases and a full 6-month follow-up for each individual enrolled in the trial.

This study has several limitations. One of them is the retrospective character of this trial. Next, there was no control group, as it was strongly recommended to apply prophylactic of Evusheld to all patients with hematological malignancy, as it was agreed that withholding these potentially life-saving medications would not have been ethical. The neutropenia or lymphopenia status was not assessed. SARS-CoV-2 strains were not genotyped in the study population, so it can only be assumed that specific SARS-CoV-2 variants of concern were present using the data of the Institute of Health Information and Statistics of the Czech Republic and the National Institute of Public Health. Moreover, it is difficult to distinguish between reinfection and reactivation of the virus, which is very commonly described, especially in patients treated with anti-CD20 drugs.

The pivotal PROVENT trial was conducted when Alpha, Beta, Gamma, and Delta variants of concern (VOC) were predominant (1). Against these VOC, Evusheld retained full or nearly full neutralization activity. However, with the ongoing mutation of the SARS-CoV-2 spike protein and its receptor binding domain (RBD), a target for numerous mAbs, the neutralization activity of these mAbs is lower or even zero [20]. In fact, the original omicron BA.1 VOC possesses 15 mutations in the RBD that lead to the evasion of RBD-targeted antibodies, with more unique mutations in subsequent subvariants, including BA.2, BA.4/5, BQ.1, and XBB lineages [21–23]. Based on in vitro studies, Evusheld retains its neutralization activity against subvariants BA.2 and BA.4/5 but is defenseless against the attacks of BQ.1 and XBB.1.5 subvariants [23–26]. More recently, the neutralization activity of Evusheld against omicron subvariants was tested from serum samples of healthy individuals. This study confirmed significantly decreased neutralization activity against BF.7, BQ.1, and XBB1.5, with XBB1.5 showing the strongest escape activity among the subvariants [27]. To prevent exposure of patients to the possible adverse effects of Evusheld, the American FDA limited its use in January 2023 only when the combined frequency of non-susceptible SARS-CoV-2 variants nationally is less than or equal to 90% [28].

**Fig. 2:**
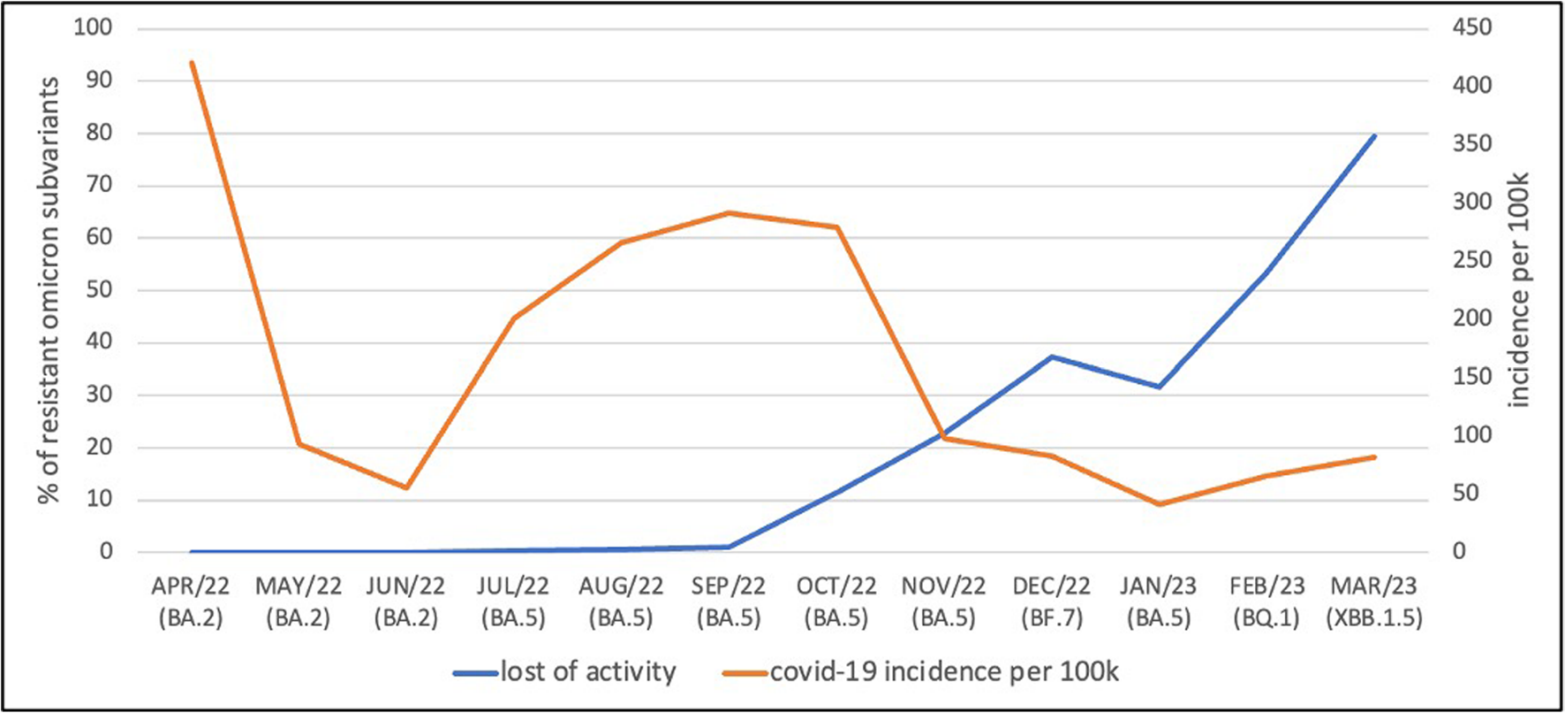
Epidemiology: the total percentage of omicron subvariant activity

According to data based on whole-genome sequencing from the samples of the general population infected with SARS-CoV-2, the Omicron VOC and its subvariants (BA.2, BA.5, BF.7, BQ.1, and XBB.1.5) were the most prevalent consecutively in the Czech Republic between May 2022 and March 2023 (Fig. 2). Figure 2 also shows the total percentage of omicron subvariants activity. An increasing amount of resistant omicron subvariants was observed, reaching 80% in March 2023. Nonetheless, higher rates of COVID-19 were not observed in the course of the study, nor were any of the fatal cases (those that happened between June 22 and October 22). With the overall amount of 96 patients who had a SARS-CoV-2 positive test during the 6-month follow-up after Evusheld administration, of whom only 2 died directly and 1 in association with COVID-19, we assume that subvariants of omicron resistant to Evusheld did not significantly affect the primary results of this study. On the other hand, the relatively low incidence of COVID-19 per 100,000 inhabitants in the Czech Republic between May 2022 and March 2023 must be considered (Fig. 2).

Overall, a 16% incidence of SARS-CoV-2 positive cases was observed throughout the 6-month follow-up after Evusheld administration as a pre-exposure prophylaxis in patients with hematology malignancy. Symptomatic COVID-19 was observed in 13.8% of patients, and more importantly, only 1.7% experienced severe course of COVID-19, with 2 cases leading to death. From our point of view, this is the most significant finding, because the primary goal of prophylactic administration of anti-SARS-CoV-2 mAbs is to prevent severe cases of COVID-19, not to prevent infection itself. We believe that to reduce the risk of COVID-19 deaths, clinicians are justified in using all available treatment possibilities, including the administration of prophylactic mAbs, vaccination, and specific antiviral treatment, especially to immunocompromised hematology patients.

## Conclusion

In conclusion, this study provides additional proof of the concept about utilizing long-lasting monoclonal antibodies against SARS-CoV-2 virus as a prevention against symptomatic COVID-19 in patients with hematological malignancies. It was demonstrated that Evusheld provides effective protection from severe COVID-19 in the most high-risk group of patients with minimal adverse effects. Previous treatment with anti-CD20 monoclonal antibody was a risk factor for COVID-19 infection, while vaccination or presence of antibodies did not reduce an incidence of COVID-19. Given the loss of neutralization activity of Evusheld and other mAbs against subsequent omicron subvariants, novel drugs are under development as the virus is still one step ahead.
